# Feature-Based Attentional Weighting and Re-weighting in the Absence of Visual Awareness

**DOI:** 10.3389/fnhum.2021.610347

**Published:** 2021-01-29

**Authors:** Lasse Güldener, Antonia Jüllig, David Soto, Stefan Pollmann

**Affiliations:** ^1^Department of Experimental Psychology, Otto-von-Guericke-University, Magdeburg, Germany; ^2^Ikerbasque, Basque Foundation for Science, Basque Center on Cognition, Brain, and Language (BCBL), San Sebastian, Spain; ^3^Department of Experimental Psychology and Center of Behavioral Brain Science, Otto-von-Guericke-University, Magdeburg, Germany; ^4^Beijing Key Laboratory of Learning and Cognition and School of Psychology, Capital Normal University, Beijing, China

**Keywords:** feature-based attention, attentional weighting, visual selection, cognitive control, unconscious

## Abstract

Visual attention evolved as an adaptive mechanism allowing us to cope with a rapidly changing environment. It enables the facilitated processing of relevant information, often automatically and governed by implicit motives. However, despite recent advances in understanding the relationship between consciousness and visual attention, the functional scope of unconscious attentional control is still under debate. Here, we present a novel masking paradigm in which volunteers were to distinguish between varying orientations of a briefly presented, masked grating stimulus. Combining signal detection theory and subjective measures of awareness, we show that performance on unaware trials was consistent with visual selection being weighted towards repeated orientations of Gabor patches and reallocated in response to a novel unconsciously processed orientation. This was particularly present in trials in which the prior feature was strongly weighted and only if the novel feature was invisible. Thus, our results provide evidence that invisible orientation stimuli can trigger the reallocation of history-guided visual selection weights.

## Introduction

For survival in an unstable and uncertain world, it is crucial to detect contextual regularities, but also to adapt quickly when they change. Since such contextual changes may be complex and occur very rapidly, the question arises as to whether attention shifts in response to environmental changes are contingent on visual awareness. Previous studies examined the effect of exogenous invisible cues on the deployment of external visual selective attention, suggesting that subliminal spatial cues can capture attention and facilitate task performance at the cued location (McCormick, 1997; Mulckhuyse et al., 2007; for a review see Mulckhuyse and Theeuwes, 2010), that the association between a subliminal cue and a visible target can be learned implicitly (Lambert et al., 1999) and that subliminal stimulus can even induce cognitive control processes like response inhibition or task-switching effects (Lau and Passingham, 2007; Van Gaal et al., 2008, 2010; Farooqui and Manly, 2015). This notion is further supported by evidence from clinical studies in “blindsight” patients, which indicate that visual cues presented in the patient’s blind field are still capable of directing spatial attention (Kentridge et al., 1999).

It is, however, less clear whether feature-based attention can be redirected towards a novel feature (feature-based attentional re-weighting) in response to changes in unconsciously processed *targets*: according to Bundesen’s theory of visual attention (TVA, Bundesen, 1990), the attentional selection is a mechanism that operates in the service of perceptual categorization, i.e., by aiding the selection of a potential target item within a distractor display (“filtering”), or the discrimination of features in single items (selection of categories, “pigeonholing”). The processing speed for this visual selection depends on both the attentional weight and the perceptual decision bias. In theory, the attentional weight relies on the sensory evidence indicating the category a certain stimulus belongs to (“bottom-up”), and the goal-relevance of that category i.e. the importance of attending to a certain stimulus category (“top-down”; Bundesen, 1990). Thus, the weaker the sensory evidence is, the more the attentional weighting should rely on the “top-down” mechanism (the importance to attend to this category). Based on the TVA’s assumption a “top-down” driven attentional bias (i.e., the goal-relevance) on selection is predicted especially for invisible non-consciously processed visual stimuli because the sensory evidence that could support visual selection in a bottom-up fashion (i.e., the saliency of the stimulus) is very limited if the stimulus is only unconsciously perceived. Importantly, evidence is still missing as to whether such a feature-based selection bias can be elicited for subliminal, unconsciously processed stimuli and whether it can be reweighted flexibly in response to feature changes of the unconsciously processed stimulus.

Later accounts of visual attention criticize the dichotomy of bottom-up vs. top-down attentional weighting and propose to include a history-driven weighting of attentional selection (e.g., Awh et al., 2012; Theeuwes, 2018, 2019) to better incorporate empirical evidence showing that not only can stimulus saliency and internal goals (volitional control) bias attentional selection but the “history” of former attention deployments driven by e.g., reward, intertrial priming, or statistical learning (Awh et al., 2012) can also have an influence. For consciously perceived visual stimuli, such *history-driven* attention weighting effects have been observed in singleton search tasks. For instance, repeated presentation of the same target-defining dimension leads to response time benefits and associated activation changes in dimension-specific visual processing areas (Pollmann et al., 2006) that were interpreted as evidence for an attentional weighting of the target-defining dimension (Müller et al., 1995; Liesefeld et al., 2018). In contrast, when the target-defining dimension changes, e.g., when the target was defined by a singleton color in recent trials and then is defined by a singleton motion direction, response time costs are observed, as would be expected when attention needs to be reweighted to the new target-defining dimension. These reweighting processes occur incidentally, in the absence of an explicit instruction to attend to the new target-defining dimension (Müller et al., 2004). Furthermore, a comparable spatial attention weighting pattern is observed when implicitly learned target-distractor configurations change in the contextual cueing paradigm (Manginelli and Pollmann, 2009; Pollmann and Manginelli, 2009). When attention-weighting processes occur in the absence of explicit task demand and even after changes of implicitly learned configurations, the next question would be whether attentional reweighting can also occur as an adaptive adjustment to unconsciously perceived stimulus changes.

Therefore, this study addressed two key questions. First, we asked whether the repeated presentation of an invisible target feature can lead to a temporally persisting attentional selection bias. The second question was how flexible this attentional bias is, i.e., whether a novel invisible target can trigger the reweighting of visual attention to the new target feature in the absence of awareness. Peremen et al. (2013) studied the relation of intertrial feature priming and visual awareness during a letter search task. They reported that the repetition of the target shape speeded visual search only when the target in the prime display had been consciously perceived. Yet, it remains unknown whether unconscious reweighting of visual selection can occur for simpler orientation stimuli such as Gabor patches (Rajimehr, 2004). We also considered a different task setting in which the selection task occurred at a fixed attended location throughout the trials. In all previous studies, attention-weighting effects were examined in multi-item displays and search tasks for a singleton target. Our paradigm does not involve spatial shifts of attention but rather a process of visual selection in which the same spatial location is always attended.

Specifically, our paradigm involved an orientation discrimination task based on a central masked bar stimulus. Volunteers were instructed to discriminate whether the target stimulus was vertical or tilted irrespective of the specific direction of tilt. They had to make no further distinction between the two tilted orientations. Yet, to introduce the tilt-based attentional selection bias, we manipulated the likelihood of the two non-vertical gratings (left vs. right) so that one tilt would occur twice as often as the other. Consistent with the proportion congruency effect during priming (Bodner and Lee, 2014; Blais et al., 2016), and feature-based statistical learning (Turk-Browne et al., 2009; Chetverikov et al., 2017), an increase of the frequency at which a right or left-tilted grating appeared should result in a high selection weight for the frequent orientation indicating the importance to attend to this category. This prediction is based on the idea that the relevant feature information (e.g., the spatial orientation) of the most likely target gets represented in a form of a short-term description—the attentional template (Desimone and Duncan, 1995), to control the sensory processing so that stimuli matching the description are favored, i.e., are more readily processed in the visual system. The degree to which a stimulus matches the attentional template defines its attentional weight. Thus, Gabor patches that fit the information stored in the template receive a high selection weight, e.g., 1, while mismatching Gabor patches (infrequent and vertical) have reduced selection weights as the whole weight is thought to be a constant value: if the weight increases for one feature it decreases for another (Duncan and Humphreys, 1989). Now, concerning behavior a switch from the heavily weighted orientation to a target with a vertical or the infrequent spatial orientation should require a shift of selection weights due to the mismatch between the sensory input and the attentional template. This shift of attentional selection weights was expected to lead to slowing stimulus processing and response initiation eventually resulting in increased response latencies in such switch trials. The higher the selection bias for the Gabor patch’s orientation in the preceding trial, the more reweighting should be necessary to process and respond to a novel grating in the subsequent trial. Thus, particularly switch trials in which the prior orientation was the highly frequent tilt should show prolonged response latencies on the behavioral level, given that the increased likelihood of one orientation over the others was sufficient to induce a prior selection bias (e.g., Leber et al., 2009; Chetverikov et al., 2017). Importantly, a combination of signal detection theoretic measures (Stanislaw and Todorov, 1999) and subjective perceptual ratings (Ramsøy and Overgaard, 2004) was used to assess participants’ awareness of the stimulus to avoid potential confounds due to criterion biases in reporting (un)awareness, e.g., reports of no experience for the knowledge held with low confidence (Wiens, 2007; Soto et al., 2019). Therefore the unconscious reweighting of selection hypothesis was eventually tested by maintaining a clear separation between the measures of selective attention weighting, inferred by the pattern of response latencies, and the measures that we used to probe (un)awareness of the stimulus (objective orientation discrimination task and subjective reports). We predicted decision reaction time (RT) costs due to a change of the tilt direction. Costs should be highest if the prior orientation was the highly biased tilt, i.e., a switch from the frequent to the infrequent tilt or a vertical target, and they should occur even if the novel target is non-consciously perceived.

## Materials and Methods

### Participants

In total 21 native German students (three male) from the University of Magdeburg, Germany took part in the experiment. All volunteers were between 19 and 34 years old (*M* = 24.90 years), right-handed by self-report except for one participant, and had a normal or corrected-to-normal vision. They provided written consent and were either monetarily reimbursed (8 euros per hour) or received course credits for the 2 h of participation. In two sessions an error in the response collection occurred and the respective participants were removed from the analysis. Another volunteer interrupted the session at an early stage and was thus excluded. During data analysis, five other participants were identified to have more than 40% missing responses during the 1.5 s response deadline (see below) and were thus excluded from RT analysis.

### Apparatus and Stimuli

The stimulus display and responses were controlled with the Python toolbox “Psychopy” (Peirce, 2007; Peirce et al., 2019). The stimuli were presented on a 24′′ Samsung monitor (1,920:1,080 resolution, 60 Hz refresh rate). All participants were placed 50 cm away from the screen. Stimuli were Gabor gratings with an individually calibrated contrast (see “Experimental Task and Procedures” section) centrally presented on a gray background subtending 3.4° visual angle. Its spatial frequency was 3.7° cycles per degree. The patch’s orientation was either vertical (180°), 165°, 150°, or 135° if it was a left-tilted, non-vertical Gabor patch, and 195°, 210°, or 225° if it was a non-vertical patch tilted to the right. To further reduce the visibility of the Gabor patch we used a circular backward mask of black and white random dots (3.4° visual angle).

### Experimental Task and Procedures

#### Threshold Determination

A session started with a staircase procedure to calibrate the stimulus’s luminance contrast rendering its orientation invisible. Gabor patches occurred centrally on the screen for 33.33 ms (i.e., the grating was presented for two frames, each of which had a minimal presentation duration of 1/60 ms) and were directly followed by the mask for 350 ms. If participants saw the grating’s orientations, they were to respond by pressing the “up”-key, while the “down”-key was to be pressed if they did not see the orientation. In the main experiment volunteers were to rate their subjective visibility of the target at the end of each trial using the four-point perceptual awareness scale (PAS): (1) “did not see anything at all,” (2) “saw a brief glimpse without seeing the orientation,” (3) “had an almost clear image of the stimulus,” and (4) “saw the stimulus and its orientation” (Ramsøy and Overgaard, 2004). During initial calibration, participants were thus instructed to report no experience of the stimulus only if they did not see anything at all which corresponded to the first point of the PAS. Conversely, they were asked to indicate an aware response in trials where a brief glimpse or a more stable percept of the Gabor was experienced corresponding to the remaining three points of the PAS. The stimulus’ luminance contrast was decreased following an aware response and increased following an unaware response. All participants did 90 trials (30 trials for each of the three orientations). The final threshold luminance was defined as the mean luminance contrast across the last 10 trials of the staircase.

Next, participants performed one block of training under experimental conditions consisting of 36 practice trials. Here the luminance contrast obtained after the first staircase procedure was used for the contrast value of the training stimuli. The practice unit was followed by a second calibration conducted according to the same protocol as the first staircase procedure. Eventually, the second recalibration provided the threshold value for the luminance contrast used in the main task.

#### Task

**Figure 1:**
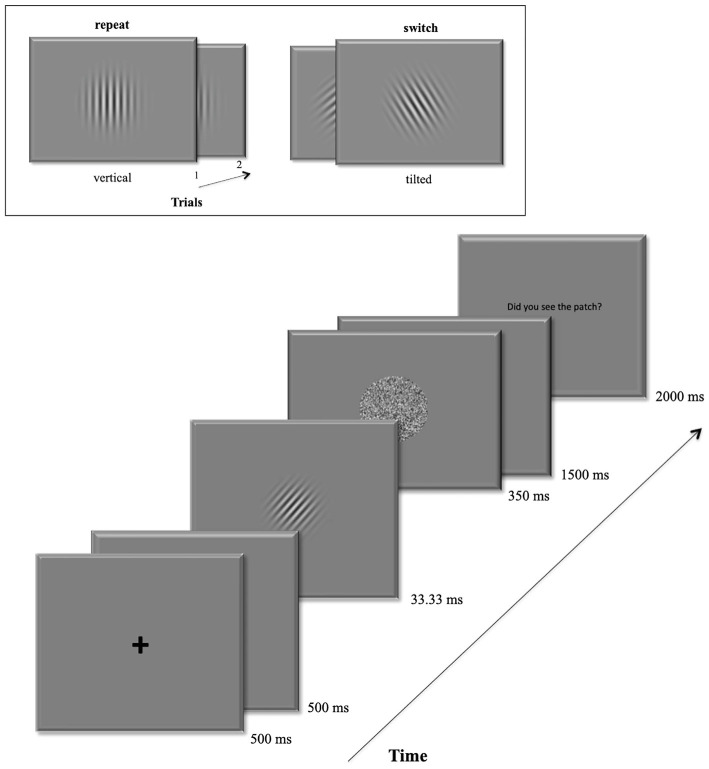
Example of a trial sequence. The box at the top shows an example of the repeat condition (left): a vertical target grating in the first trial is followed by another vertical grating in the second trial. On the right it shows an example for the switch condition: the left-tilted target grating is followed by a right-tilted grating in the next trial.

In the main experiment, volunteers were asked to perform an orientation discrimination task based on masked Gabor patches. The start of a new trial was signaled by a central fixation remaining 500 ms on the display followed by a blank screen for another 500 ms duration. Then the target Gabor patch occurred at the screen center for 33.33 ms. A pattern backward mask (Breitmeyer and Ogmen, 2000) followed immediately for 350 ms. In the following 1.5 s participants were to give their discrimination response. At the trial’s end, they were eventually prompted to rate the visibility of the Gabor patch using the keys 1–4 within the next two 2 s. All trials were separated by inter-trial-intervals (ITI) with varying durations (1.5–3.5 s) following a logarithmic distribution. Figure 1 depicts a detailed trial sequence.

### Design

To facilitate the occurrence of a tilt-based attentional selection bias, we introduced uneven proportions of the two non-vertical gratings (left vs. right). Consistent with feature-based statistical learning (e.g., Turk-Browne et al., 2009; Chetverikov et al., 2017), the relative increase of the frequency at which a right or left-tilted grating appeared was expected to strongly weight attentional selection for this orientation. Its higher likelihood should increase the importance of attending to this feature, resulting in a high selection weight. At the same time, the selection weight for the other two orientations (vertical and the infrequent tilt) should be reduced (Bundesen, 1990). Eventually, switches away from the heavily weighted tilt were expected to result in a significant increase in volunteers’ response times. Therefore, for a block of 36 trials, we chose 12 vertical targets (~33%), and used uneven proportions of the two tilts, with 18 trials (50%) and six trials (~16%), respectively. This way, each block was either left—(75% of all non-vertical trials were left-tilted) or right—weighted (75% of all tilt trials were right-tilted). The actual presentation of the three orientations was randomized within a single block.

The first 11 participants performed 14 blocks in the main experiment (504 trials). The eighth subject, however, interrupted the session after 12 blocks were completed. Subjects 12–21 completed 10 blocks (360 trials) as this amount of trials turned out to be sufficient to obtain enough trials for each awareness level (AL) while avoiding growing weariness that was reported by subjects completing 14 blocks.

### Statistical Analysis

Sensitivity and response bias measures were calculated using custom-made Python code (Version 2.7). All statistical analyses were carried out with R (Version 3.5, R Core Team, 2014). For the Bayes factor (BF) analysis (Rouder et al., 2009) we used JASP (JASP Team, 2019).

#### Subjective Awareness

For each participant, the number of trials for each subjective AL was counted using the trial-by-trial PAS-rating.

#### Discrimination Performance

To examine whether the participant’s ability to correctly discriminate between vertical and non-vertical gratings depends on the level of subjective awareness, we firstly determined individual response bias and perceptual sensitivities using signal detection theory (Stanislaw and Todorov, 1999; Macmillan and Creelman, 2004). Group-effects were subsequently assessed for each level of subjective awareness using BFs since it was required to prove the *absence of sensitivity* (H0; Gallistel, 2009; Dienes and Mclatchie, 2018). A BF_(10)_ provides moderate evidence for H0 (e.g., *A’* = 0.5) if it stands between 0 and 0.33, anecdotal evidence if it stands between 1/3 and 1, and evidence for H1 (*A’* > 0.5) if it exceeds 1 (Dienes and Mclatchie, 2018), with a BF_(10)_ between 1 and 3, 3 and 10, 10 and 30, 30 and 100 and >100 providing anecdotal, moderate, strong, very strong, and extreme evidence, respectively, for H1 (Jeffreys, 1998; Quintana and Williams, 2018).

Under Yes/No-conditions *A’* and the *criterion location* (*C)* were calculated to determine perceptual sensitivity and bias: we calculated *false-positive rat*es [FPR = False alarms/(False Alarms + Correct Rejections)] and *hit rates* [TPR = Hits/(Hits + Misses)] defining a *hit* as the correct report of a non-vertical orientation when the Gabor’s orientation truly was tilted; *false alarms* were defined as tilt response for vertical gratings. We used the following formulas to calculate the non-parametric response bias and sensitivity (Stanislaw and Todorov, 1999):

C=−[Z(TPR)+Z(FPR)]/2

$$A^{\prime }=\text{0.5}+\left|sign\left(TPR-FPR;\left(TPR-FPR\right)\text{2}+\left|TPR-FPR\right|/\left(\text{4max}\left(TPR,FPR\right)-\text{4}*TPR*FPR\right)\right)\right|$$

Values of C around 0 indicate unbiased discrimination performance. A liberal decision criterion favoring yes-responses (i.e., reporting a non-vertical grating) leads to values of *C* < 0, while positive values occur if participants are biased to report a vertical target. If volunteers possess perfect sensitivity at discriminating the target orientations, A’ appears to be equal to 1 and it decreases to 0.5 if the sensitivity diminishes (Stanislaw and Todorov, 1999).

#### Analysis of RT Data

We used the packages lme4 (Bates et al., 2015) as well as lmerTest to make use of a linear mixed model (LMM) analysis. As the data was unbalanced due to the variations in the subjective awareness ratings (PAS) that lead to uneven numbers of trials across the four levels of visual awareness, LMMs were chosen over custom repeated measures ANOVAs to analyze the RT (e.g., Avneon and Lamy, 2018). Since all cases with missing data would be excluded in a repeated-measures ANOVA, the LMM approach is the better means to make use of all available data in the face of an unbalanced design (Magezi, 2015). Only RTs of trials with correct responses entered the analysis after each participant’s individual outliers (mean RTs ± 3 *SD*) were removed.

Before assessing the significance of the fixed effects, we determined the random effect structure of the final model with likelihood ratio tests (i.e., comparisons of models differing in their random effect structure). Importantly, we did not use likelihood ratio tests to compare models with differences in their fixed effects as these were already determined by the design (see below). Once the final model for analysis was fully defined, we fitted this model with the RT data using a restricted maximum likelihood estimation (REML) and tested the statistical significance of the fixed effect predictors with a type III ANOVA with F-statistics as implemented in the *lmer* function of the *lme4* package (Version 1.1–23; Richardson and Welsh, 1995; Bolker et al., 2009; Luke, 2017; McNeish, 2017). The *p*-values were calculated using Satterthwaite approximations to degrees of freedom with the *ANOVA* function of the package *lmerTest* (Version 3.1-2, Kuznetsova et al., 2017). We chose the ANOVA approach to test the statistical significance of the fixed effects as this approximation is thought to be producing acceptable Type 1 error rates even for small samples while the use of model comparisons (likelihood ratio tests) is not recommended to test fixed effects because they appear to be anti-conservative (Pinheiro and Bates, 2000; Bolker et al., 2009; Luke, 2017). *Post hoc* tests (least squared means of the contrasts with Bonferroni correction) were performed using the R package *emmeans* (Version 1.4.7). Finally, we used the R function *r.squaredGLMM* as implemented in the R package *MuMin* to calculate the marginal *R* squared ($R_{m}^{\text{2}}$) and conditional *R* squared ($R_{c}^{\text{2}}$) to obtain standardized effect sizes. $R_{m}^{\text{2}}$ is interpreted as the variance explained by the fixed effects of awareness and switch and $R_{m}^{\text{2}}$ gives the variance explained by all fixed and random effects (Johnson, 2014).

The main goal we pursued in the study was the examination of whether a changing orientation from one trial to another *(switch)* affected participants’ responses: we predicted a switch-related slowing of RTs compared to trials in which the orientation remained unchanged *(repeat)*. Thus, the switch of orientations (switch vs. repeat) constituted the first fixed effect predictor in the LMM. RTs were also expected to decrease with increasing visual awareness: the more the participants saw, and the more confidently they should perform at categorizing the stimulus orientation, the faster they should be at responding to the grating’s orientation. Therefore, visual awareness was defined as the second fixed effect predictor of the basic model. Finally, to make allowance for a possible interaction between the two fixed effects we included the interaction term of switch and awareness into the final LMM. Regarding interindividual baseline differences in response latencies, we also defined a by-subject random intercept accounting for non-independency of single subjects’ data. Thus, the basic model was formalized as *RT ~ switch + awareness + switch:awareness (1 | subject)*.

In this model, however, the full random effect structure still needed to be determined. Therefore, we next used model comparisons based on likelihood ratio tests (χ^2^) with the *ANOVA* function of the *lme4* package (Baayen et al., 2008) to assign the full random effect structure (Barr et al., 2013) of this basic model. Defining the random effect structure is important to balance between the type I error rate that inflates if the random effect structure of an LMM is underspecified (Barr et al., 2013), and the model power that suffers if the random effect structure is more complex than the given data (Matuschek et al., 2017). The method of model comparisons based on likelihood ratio tests compares to the procedure of a hierarchical regression in which relevant predictors are added to the regression model and kept if they significantly improve the model fit (changes in *R^2^*). Likelihood ratio tests are deemed to be appropriate to formally define the random effect structure of an LMM even if the sample size is small (Baayen et al., 2008; Bolker et al., 2009). Using this method, we tested the basic model containing only a by-subject intercept against alternative models containing an additional by-subject random slope for awareness and/or a by-subject random slope for the switch. The details of this analysis are reported in the Supplementary Material. Importantly, we used the likelihood ratio tests only to determine the random effect structure of the final model that we used to fit the RT data with, while the significance of the fixed effects (i.e., the hypotheses testing) was assessed using the type III ANOVA with Satterthwaite approximations to degrees of freedom (Luke, 2017). Based on the model comparisons we included a by-subject random slope for awareness to model potential by-subject heteroscedasticity concerning awareness [i.e., allowing uneven variances across the levels of the fixed effect awareness (Baayen et al., 2008)]. Eventually, the final model for significance testing was defined as *RT ~ switch + awareness + switch:awareness (1 + awareness | subject)*.

The final LMM with the structure outlined above was applied in two RT models: In the first model (average RT model) we included all possible orientation changes in the switch condition. In the second model (weighted RT model) the switch condition contained only those switch trials in which we expected the highest RT costs to occur: the frequency differences between the three orientations were expected to boost the selection weight for the highly frequent non-vertical orientation (either left or right). Consequently, re-weighting to the infrequent non-vertical orientation should be associated with more pronounced switch costs than vice versa. The same was predicted for changes away from the heavily weighted to the vertical orientation requiring stronger attentional re-weighting. However, switches away from the low-frequent tilted orientation to vertical should lead to less prominent RT costs because the attentional selection weight for this tilted orientation is weaker, facilitating the shift of attentional resources towards the novel target orientation. Hence, these trials were not included in the weighted RT model. We separately report the results for the LMM analyses for the average and the weighted RT model.

## Results

### Subjective Awareness

In the majority of trials, participants’ subjective awareness of the to-be-discriminated orientation was low (AL2, 25.94%), or reported experience was fully absent (AL1, 37.70%). In about 26.80% of all trials, subjects reported an almost clear perception of the grating (AL3) and its orientation. In only 9.54% of all trials did they clearly see the grating and its orientation (AL4). Due to the low number of these AL4 trials, we excluded them from the following analyses. A detailed summary of the number of trials for switch and repeat trials for each level of subjective awareness is reported in the Supplementary Material.

After the experimental session, each volunteer was asked to report whether any differences in the frequencies of the stimulus orientation had been noticed. The majority of subjects reported that more tilted than vertical orientations had been presented but none of the participants noticed the block-wise changing frequency difference for the tilted orientations (left vs. right).

### Objective Discrimination Ability and Subjective Awareness Concordantly Diminish

Signal detection analyses revealed that on trials with almost full (AL3) and partial awareness (AL2) participants’ perceptual sensitivity was significantly above chance. Bayes-factors provided extreme evidence that sensitivity (A’) was greater than 0.5 in AL3, BF_(10)_ > 100, 95% CI (0.733, 0.845), and AL2 trials, BF_(10)_ > 100, 95% CI (0.672, 0.750) (Quintana and Williams, 2018). The mean A’ of 0.789 ± 0.026 (SE) in AL3 trials was 7.6 times more likely to be greater than the mean A’ of 0.711 ± 0.018 in AL2 trials, BF_(10)_ AL3 > AL2 = 7.608 (Quintana and Williams, 2018). On unaware trials (AL1), however, we observed a mean A’ of 0.516 ± 0.030 that was more likely to be equal to 0.5 with moderate evidence for the H0, BF_(10)_ = 0.317, 95% CI (0.451, 0.582) (Quintana and Williams, 2018), indicating the absence of perceptual discriminability of the gratings’ orientation.

In contrast to the perceptual sensitivity analyses, individual response biases remained unaffected by changes in subjective awareness. In none of the four ALs did we find clear evidence for a more liberal or more conservative response criterion to report a non-vertical orientation, than a C around 0. BFs were rather in favor of the null hypothesis indicating that the mean C of −0.188 ± 0.173 in AL1 trials was more likely not different from zero, yet with only anecdotal evidence for the H0, BF_(10)_ = 0.457, 95% CI (−0.560, 0.188) (Quintana and Williams, 2018). The same was true for the mean C of −0.073 ± 0.106 in AL2 trials, BF_(10)_ = 0.342, 95% CI (−0.303, 0.157), and for a mean C of 0.089 ± 0.123 in AL3 trials, BF_(10)_ = 0.349, 95% CI (−0.179, 0.357) (Quintana and Williams, 2018).

To examine variations in the response criterion location (C) across the three levels of subjective visual awareness, we made use of LMM to optimally deal with the unbalanced data set (Magezi, 2015). Since we aimed to assess the absence of variations in C across the three levels of subjective awareness, we conducted a Bayesian-based LMM analysis using the R package BayesFactor to obtain a Bayes factor (BF_(10)_) directly proving the null hypothesis (Morey and Rouder, 2018): first we constructed a null model in which only a by-subject random intercept was included assuming that variations in C relied on interindividual differences only [C ~ 0 + (1| subject)]. Next, we constructed an alternative model in which the subjective awareness reports (awareness ratings 1–3) served as a single fixed effect explaining variance in C in addition to the by-subject random intercept [C ~ awareness + (1 | subject)]. Using the lmBF function we then calculated BFs for each model and compared the two models by dividing the BF of the model that included awareness as a fixed effect by the BF of the null model. The analysis resulted in an inconclusive BF_(10)_ of 0.58 providing weak evidence for the absence of variation in C across the three levels of subjective awareness (Quintana and Williams, 2018).

**Figure 2:**
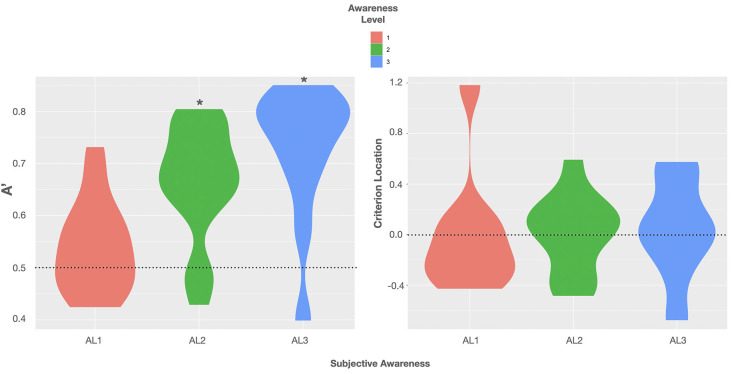
Violin plots of group distributions of sensitivity A’ (left) and the criterion location (response bias; right) for each level of subjective awareness [perceptual awareness scale (PAS) ratings AL1–AL3]. Left: the agreement of the objective and subjective measure of visual awareness is indicated by moderate evidence for the absence of sensitivity on trials rated as subjectively unaware; BF_(10)_ < 0.33. Black asterisks = BF_(10)_ > 100 indicating extreme evidence for A’ being truly > 0.5. Violin plots use density curves to depict distributions of numeric data. The width corresponds with the approximate frequency of data points in each region. The lower and upper limits of each plot are determined by the minimum and maximum values.

Descriptive data of sensitivity and bias measures are reported in Table 1. The data showed that the performance of at least two subjects was highly biased in trials rated as fully unaware with a shift in C of +1 *SD* and −1 *SD*, respectively. A graphic illustration of the relation between the objective measures of awareness and the subjective measure is shown in Figure 2 depicting violin plots of A’ and C for each level of subjective awareness. In sum, these results show that the ability to distinguish the two types of orientation (non-vertical vs. vertical) strongly depended on the subjective visibility and fully diminished on subjectively unaware trials. In contrast, there was no clear evidence of a response bias, regardless of the level of subjective awareness. Importantly the absence of variation in volunteers’ response bias likely suggested that perceptual decision criteria were not dependent on the awareness reports and that variations in participants’ perceptual sensitivity regarding the stimulus orientation could thus not be caused by variations in the response bias.

**Table 1:** Average sensitivity (A’) and criterion location (bias, C) for the orientation discrimination task for each level of subjective awareness.

	Awareness					
	Level 1		Level 2		Level 3	
	*A’*	*C*	*A’*	*C*	*A’*	*C*
M	0.515	0.048	0.670	−0.093	0.723	−0.152
SD	0.091	0.438	0.110	0.402	0.135	0.527

In the signal detection analysis outlined above, we assumed a binary yes/no task setup. However, as we deployed left and right-tilted gratings next to vertical ones, subjects were to sort three possible stimulus types into two categories. Moreover, we presented left- and right-tilted Gabors with varying angles so that subjects needed to map different stimuli to the same response. Hence, a classification scenario may better fit the scenario (Snodgrass et al., 2004). Importantly, such a setup requires the implementation of two rather than one decision criterion increasing the decision uncertainty, and the proportion of correct responses [i.e., *proportion correct*, *p(c)*] is then used to measure volunteers’ classification sensitivity (Macmillan and Creelman, 2004, p. 190–191). Hence, our sensitivity measure may not be exhaustive of all the information that the subject could hold, meaning that actual sensitivity on unaware trials could be higher than we measured.

Thus, we additionally calculated p(c) for each level of subjective awareness (AL1–AL3): p(c) can be defined as the prior probability of a positive stimulus (i.e., non-vertical grating) times the conditional probability of a positive response given a positive stimulus (i.e., a non-vertical response for a non-vertical target) added to the product of the prior probability of the negative stimulus (i.e., vertical) times the conditional probability of a negative response given a negative stimulus (Swets, 2014, p. 4). In other words, p(c) is found by using the presentation probability of the two non-vertical targets as weights for the hit rate and adding this to the product of the 1-False alarm rate (i.e., correct rejection rate) and the presentation probability of the vertical target [i.e., p(c) = (8/36)*H + (16/36)*H + (12/36)*(1-F); Macmillan and Creelman, 2004, p. 89].

**Figure 3:**
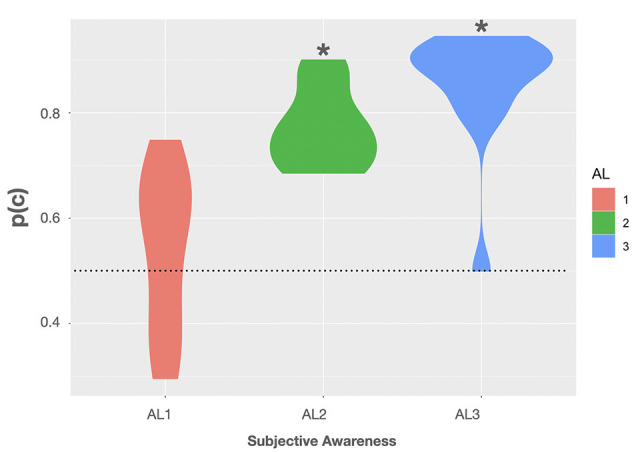
Violin plots show the observed proportions of correct responses [p(c)] as a function of subjective awareness (AL1–AL3). Black asterisks indicate that testing p(c) on group level against a theoretical chance level of 0.5 (dotted line) resulted in a Bayes factor (BF) providing extreme evidence for p(c) being greater than 0.5 (BF_(10)_ > 100). Violin plots use density curves to depict distributions of numeric data. The width corresponds with the approximate frequency of data points in each region. The lower and upper limits of each plot are determined by the minimum and maximum values.

Using this formula, we observed a mean p(c) of 54 ± 4.2% (*SE*) in trials rated as fully unaware. Here the BF was rather inconclusive as to whether p(c) was different from the 50% chance level with only anecdotal evidence for the H0, BF_(10)_ = 0.409, 95% CI (44.9, 63.1) (Quintana and Williams, 2018). In AL2 trials the mean p(c) on group level was 77.1 ± 2% associated with a BF providing extreme evidence that p(c) was truly above chance, BF_(10)_ > 100, 95% CI (72.6, 81.5) (Quintana and Williams, 2018). In trials rated as almost fully aware (AL3) we observed a mean p(c) of 85.4 ± 3.2%. Here the BF again provided extreme evidence for p(c) to be greater than 50%, BF_(10)_ > 100, 95% CI (78.4, 92.4) (Quintana and Williams, 2018). Violin plots show the observed p(c) as a function of subjective awareness in Figure 3. For more transparency, we additionally included accuracy data obtained in the experimental task, as well as the average rates of hits (H), false alarms (FA), correct rejections (CR), and misses (M), and the mean number of hit, false alarm, miss, correct rejection trials in the Supplementary Material.

Taken together, using p(c) as a measure of volunteers’ perceptual sensitivity did not change the conclusion that participants’ classification ability was at chance in trials rated as subjectively fully unaware, while they showed considerable classification sensitivity in trials with residual and almost full subjective awareness. Importantly, the above measures are representative and exhaustive of the critical target feature that is relevant for the task (i.e., orientation; Snodgrass et al., 2004). However, additional experimentation could be performed employing an even more stringent detection threshold in which one’s sensitivity to detect the presence of any grating is null.

### RT Data

We analyzed volunteers’ RT data to test whether the latency of the manual responses slowed down during (unconscious) changes in the target orientation compared to repeating target orientations which would suggest a reweighting of attentional selection weights. Individual outliers (*M* ± 3 *SD*) were removed before the LMM analysis. We conducted the same LMM analysis for two RT models. While in the first *average* RT model the switch condition comprised all possible orientation changes, the second *weighted* RT model included only switch trials in which the prior target orientation was associated with a high selection weight (i.e., changes away from the most frequent tilt). Descriptive mean RTs and *SE*s of both models for switch vs. repeat trials for each level of awareness are summarized in Table 2.

**Table 2:** Mean (M) reaction times (RTs) and standard deviations (SD) for the switch and repeat trials for each level of subjective awareness summarized of (A) the average RT model comprising the mean of all switch trials and (B) the weighted RT model including only weighted switch trials.

	Awareness					
	Level 1		Level 2		Level 3	
	Switch	Repeat	Switch	Repeat	Switch	Repeat
(A) Average switch
M	1.110	1.068	0.945	0.917	0.889	0.869
SD	0.225	0.231	0.182	0.212	0.130	0.128
(B) Weighted switch
M	1.177	1.068	0.947	0.917	0.879	0.869
SD	0.242	0.231	0.194	0.212	0.129	0.128

To begin, we conducted the LMM analysis for the average RT model in which the mean of the switch condition included all possible switch trials. Visual inspection of residual plots did not reveal any obvious deviations from homoscedasticity nor normality. Estimated RTs appeared to be sensitive to changes in the level of visual awareness indicated by the significant fixed effect of awareness, *F*_(2,11.055)_ = 9.6740, *p* = 0.0037. In line with our predictions, the *post hoc* tests showed that RTs (averaged across the conditions switch and repeat) in AL1 trials were 157.6 ± 41.6 ms slower compared to AL2 trials, *t*_(12.00)_ = 3.783, *p* = 0.0078, 95% CI (41.8, 273.5), and 225.6 ± 54 ms slower compared to AL3 trials, *t*_(11.87)_ = 4.177, *p* = 0.0039, 95% CI (75.2, 376.1). RTs in AL2 and AL3 trials did not differ significantly, *p* = 0.3678, 95% CI (−46.0, 182.0). Thus, RTs of the average RT model was indeed sensitive to changes in visual awareness and decreased with increasing stimulus visibility.

There was, however, no significant main effect of switch, *F*_(1,35)_ = 3.1709, *p* = 0.0836, nor a significant interaction *F*_(2,35)_ = 0.1411, *p* = 0.8689 showing that RTs appeared to be unaffected by changing stimulus orientations in this RT model. About 20% of the total variance was explained by the model’s fixed effects, *R_m^2_* = 0.1989, and 88% by the model’s fixed and random effects, *R_c^2_* = 0.8843.

Next, we used the same LMM to analyze the weighted RT model in which the switch condition comprised only switch trials where the prior orientation was the heavily weighted one. Again, visual inspection of residual plots did not reveal any obvious deviations from homoscedasticity nor normality. The LMM analysis showed, also in this model, that estimated RTs increased with decreasing visual awareness, *F*_(2,10.93)_ = 10.9895, *p* = 0.0024. The *post hoc* tests with Bonferroni correction indicated that mean RTs across both switch and repeat trials were on average about 190.5 ± 45.6 ms significantly slower in AL1 trials compared to AL2, *t*_(12)_ = 4.177, *p* = 0.0039, 95% CI (63.7, 317.3) and on average 263.1 ± 58.7 ms slower compared to AL3 trials *t*_(11.92)_ = 4.482, *p* = 0.0023, 95% CI (99.7, 426.4). Mean RTs in AL2 and AL3 trials did not differ significantly, *p* = 0.3007, 95% CI (−40.8, 185.9).

Importantly, now also a switch of the target orientation affected RTs: the analysis revealed a significant fixed effect predictor switch, *F*_(1,35.00)_ = 6.0303, *p* = 0.019. Here the *post hoc* tests suggested that only in unaware trials (AL1) were RTs in response to a novel orientations on average 109.5 ± 34.5 ms significantly slower compared to trials in which the orientation was repeated, *t*_(35)_ = −3.171, *p* = 0.0029, 95% CI (−179.7, −39.4). In trials with higher levels of visual awareness, switch costs were not significant, AL2, *p* = 0.4033, 95% CI (−99.4, 40.9); AL3, *p* = 0.7789, 95% CI (−83.1, 62.8). Yet, there was no significant interaction between the two fixed-effect predictors awareness and switch, *F*_(2,35.00)_ = 2.2800, *p* = 0.1172. Together, about 26% of the total variance was explained by the two fixed effects awareness and switch, *R_m^2_* = 0.2568, and about 85% was explained by all fixed and random effects, *R_c^2_* = 0.8532.

**Figure 4:**
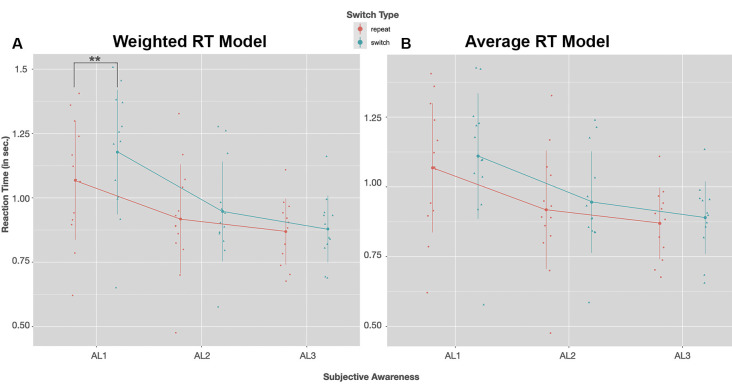
Group mean RTs (in seconds) for a switch (blue) and repeat trials (red) as a function of visual awareness (AL1–AL3); on the left **(A)** RTs of the weighted switch model, on the right **(B)** RTs of the average switch model is shown. Dots and triangles indicate individual participant data points. Vertical lines show the range of 1SD ± the mean. In both reaction time (RT) models *post hoc* tests with Bonferroni correction revealed that RTs speeded up with increasing visual awareness (AL2–AL1: *p* < 0.01, AL3–AL1: *p* < 0.01). A significant slowing of RTs in switch compared to repeat trials was observed only for the weighted model in unconscious trials (AL1); **p < 0.01 (*Post hoc* tests with Bonferroni correction).

RTs for both switch and repeat trials as a function of visual awareness for the weighted and the exhaustive RT model are plotted in Figures 4A,B. The LMM solutions for the fixed and random effects for the two RT models are given in Tables 3A,B.

**Table 3:** Fixed and random effects solutions of the linear mixed model (LMM) for the average RT model (A) and the weighted RT model (B).

Fixed effects	Estimate (in seconds)	*SE*	*t*-value	*p*-value
(A) Average switch
Awareness
AL1:	−1.06823	0.05965	17.203	1.62e-10^***^
AL1–AL2	−0.15035	0.04457	−3.241	0.00452**
AL1–AL3	−0.21491	0.05536	−3.726	0.00254**
Switch (Intercept)	−0.04209	0.02766	1.460	0.15309
AL2:
Switch—Repeat	−0.01465	0.03912	−0.359	0.72144
AL3:
Switch—Repeat	−0.02151	0.03992	−0.539	0.60840
**Random effects**	**Variance**	*SD*
Subject	0.044852	0.21148
AL1–AL2	0.017186	0.13109		
AL1–AL3	0.031373	0.17712		
Residual	0.005399	0.07348
(B) Weighted switch
Awareness
AL1 (Intercept)	−1.06823	0.06362	16.792	1.19e-10^***^
AL1–AL2	−0.15035	0.04970	−2.905	0.00896**
AL1–AL3	−0.21338	0.06106	−3.354	0.00451**
Switch (Intercept)	−0.10959	0.03316	3.171	0.00315**
AL2:
Repeat—Switch	−0.08035	0.04690	−1.644	0.10905
AL3:
Repeat—Switch	−0.09941	0.04786	−2.077	0.05408
Random effects	Variance	*SD*
Subject	0.044852	0.21178
AL1–AL2	0.017821	0.13889
AL1–AL3	0.035990	0.18971
Residual	0.007761	0.08809

Note: significance codes: ^***^p < 0.001; **p < 0.01. (A) Average RT model: fixed effect predictor switch includes all types of orientation changes. (B) Weighted RT model: fixed effect predictor switch comprises only the changes away from the heavily weighted orientation. *P*-values indicate the difference between each factor level compared to baseline (intercept). For both models: intercept switch equals the estimated mean difference of switch trials compared to repeat trials across all three levels of awareness, intercept AL1 equals the mean of all switch and repeat trials rated as subjectively unaware. Random effects AL1–AL2, and AL1–AL3 indicate the amount of variation in the fixed effect switch between the two AL1 and AL2, and AL1 and AL3, respectively.

In sum, the LMM analysis suggests that not only were RTs sensitive to decreasing visual awareness but also changes in the stimulus orientation. However, RT costs due to such changes were observed only in the weighted RT model which included only those switch trials in which the novel orientation changed away from the highly biased orientation (highly frequent tilt) fostering the conclusion that the prior visual selection bias had boosted behavioral switch costs in response to a change in the target orientation. As significant switch costs were observed in unaware trials only, the impact of the prior selection bias boosting behavioral switch costs during attentional re-selection appeared to be most prominent in the full absence of visual awareness.

Given that under unconscious conditions we had fewer trials included in the analysis, outliers could have a stronger effect on the results. Since 3 SD is not a rigid cutoff for outliers, we, therefore, repeated the analysis with a 2 SD, and 2.5 SD cutoff but the results did not change in terms of significant fixed effects. Most relevant to our research question, we found a significant switch effect for the weighted RT data model in AL1 but neither in AL2 nor in AL3 trials for all three cutoffs. We conducted further control analyses that are reported in the Supplementary Material in which we matched the number of trials between AL1, AL2, and AL3 trials by random sampling to prove that the low amount of AL1 trials could not account for the observed switch effect, and used the numbers of trials obtained for the weighted switch trials rated as fully unaware (AL1) to do a Bayesian-based prediction to show that the switch costs in the weighted RT model were not associated with individual trial numbers.

Finally, to rule out the possibility that intertrial response priming instead of attentional weighting could account for the observed switch effect, we repeated the LMM analysis for the weighted switch model after removing all weighted switch trials preceded by trials in which the orientation had been perceived consciously to some extent (i.e., AL2, and AL3 “pretarget” trials). This we did because intertrial response priming is thought to necessitate awareness of the stimulus in the preceding trial (e.g., Peremen et al., 2013). Using the same LMM we found only a marginal switch effect, *F*_(1,42.301)_ = 4.0112, *p* = 0.0516, a significant fixed effect of visual awareness, *F*_(2,14.399)_ = 17.5605, *p* < 0.001, and a significant interaction of the two fixed effects switch and awareness, *F*_(2,42.198)_ = 4.7763, *p* = 0.0134. The fixed and random effect solutions of this analysis are given in Table 4. Importantly, paired comparisons replicated our previous finding showing that unaware weighted switch trials were significantly slower compared to unaware repeat trial, *t*_(33.24)_ = −2.954, *p* = 0.0057, 95% CI (−360.5, −66.4), while there were no differences between switch and repeat trials for AL2, *p* = 0.1157, nor for AL3, *p* = 0.2304.

**Table 4:** Mixed and random effect solution after removing those trials preceding a weighted switch in which the stimulus was consciously perceived.

Weighted switch RT model with unconscious pretarget trials only				
Fixed effects	Estimate (in seconds)	*SE*	*t*-value	*p*-value
**Awareness**
AL1	1.09469	0.07955	13.761	4.01e-10^***^
AL1–AL2	−0.17603	0.07667	−2.296	0.03137*
AL1–AL3	−0.24179	0.07637	−3.166	0.00447**
Switch (Intercept)	0.21353	0.07067	3.022	0.00414**
AL2:
Switch—Repeat	−0.114745	0.09333	−1.229	0.22556
AL3:
Switch—Repeat	−0.29642	0.09740	−3.043	0.00398**
**Random effects**	**Variance**	***SD***
Subject	0.05811	0.2411
AL1–AL2	0.02811	0.1677
AL1–AL3	0.02536	0.1592
Residual	0.02416	0.1554

Note: significance codes: ^***^p < 0.001; **p < 0.01; *p < 0.05. *P*-values indicate the difference between each factor level compared to baseline (intercept). The intercept of switch type equals the estimated mean difference of weighted switch trials compared to repeat trials across all three levels of awareness, intercept AL1 equals the mean of all weighted switch and repeat trials rated as subjectively unaware. Random effects AL1–AL2, and AL1–AL3 indicate the amount of variation in the fixed effect switch between the two AL1 and AL2, and AL1 and AL3, respectively.

## Discussion

When volunteers engaged in our discrimination task of masked gratings, RTs were sensitive to orientation changes. However, significant switch costs were obtained only if the selection weight for the prior orientation was high (i.e., the highly frequent tilt) and if the novel orientation was unconsciously perceived. Importantly, our criteria for lack of awareness were based on the combination of subjective and objective measures, i.e., no experience reports and no ability to discriminate the relevant target features in a forced-choice test. To the best of our knowledge, this, therefore, is the first study investigating the effects of unaware targets on feature-based attention weighting by using a combination of objective sensitivity measures and subjective measures of visual (un-)awareness collected *during* the experimental task. This is a very important advantage for two reasons: first, to account for fluctuations of the perceptual threshold before, during, and after the actual experimental task it is extremely important to use an “online” measure of visual awareness during the task performance. This way, one ensures that the stimulus perception and the effect of the stimulus are measured in the same context (e.g., Avneon and Lamy, 2018). Second, studies that define unconscious processing only employing subjective awareness measures (e.g., Cheesman and Merikle, 1986) suffer from the criterion problem that arises when conscious knowledge is held with low confidence, hence objective measures that can ensure a clear absence of visual awareness (i.e., if d’ = 0) are critical to studying unconscious information processing, which would then be pinpointed by information-based analyses of neural measures (Soto et al., 2019). Yet, to come up with an exhaustive means that measures visual awareness and unawareness equally well, the joint use of both the objective and subjective measures seems optimal (e.g., Wiens, 2007).

Taken together, our results indicate that prior feature likelihood differences modulated attentional weighting by introducing a competitive bias favoring (i.e., increasing the selection weight for) the most likely event (i.e., frequent tilt). Only when the orientation associated with a high selection weight was present in the prior trial did attentional re-selection towards a novel target orientation result in behavioral switch costs. This was indicated by significant RT differences between stay and switch trials in the weighted model. Switch costs due to changing features within a single feature dimension may be relatively small compared to cross-dimensional switch costs (see Müller et al., 1995), which could explain why there was no switch effect in the averaged model in any level of awareness. Thus, the putatively smaller effect of within-dimensional switches may require a strong prior feature weighting to emerge.

Remarkably, the behavioral switch costs were observed only in trials reported as fully unaware, in which subjects had zero sensitivity for the stimulus orientation. According to Bundesen’s (1990) TVA, the influence on sensory processing given by an attentional template that contains goal-relevant information (i.e., history-guided) becomes particularly strong if the sensory evidence of the to-be-processed stimulus is low. In such a case there is little stimulus information that could form the selection weight in a “bottom-up” fashion so that knowledge about the importance of attending to a certain stimulus category (e.g., because this category is more likely to occur) gains influence on stimulus processing. Hence, one could predict that the behavioral effect due to attentional weighting and re-weighting should be most pronounced in unaware trials in which decision making may especially rely on implicit knowledge (i.e., prior beliefs about likelihoods, Bohil and Wismer, 2015) maintained in the form of an attentional template because the weak sensory evidence given by the unconscious stimulus does not suffice to strongly bias its selection.

Importantly, this conclusion does not imply that the information (i.e., prior beliefs about likelihoods) upon which the trial history-guided attentional selection is built is derived from invisible stimuli. Certainly, in our paradigm, a significant amount of targets were perceived partially and almost fully consciously. Thus, even if the subjects’ reports suggest that the knowledge about the likelihood differences was rather implicit, it was likely to be derived from visible stimuli. Still, a shift of attentional selection weights against a prior bias was elicited by an invisible novel target. This clearly shows that invisible feature changes can indeed trigger a shift of visual attention. Peremen et al. (2013) reported the opposite pattern of results: strong intertrial feature priming if primes and probes were consciously perceived but no such repetition effects under masking conditions. However, by using prior likelihood differences of the three orientations, we introduced a feature weighting that evidently boosted the switch effect, deliberately chose simple Gabor patches that are known to be readily processed, even if unconsciously (e.g., Rajimehr, 2004; see also Soto et al., 2011; King et al., 2016), and tested participants in a simple discrimination task in which the focus of spatial attention was always directed to the relevant location, instead of using a visual search paradigm. Importantly, the prior attention bias was essential to induce prior attentional weighting impeding consequent attentional re-weighting in response to a novel target. In conclusion, this suggests that a prior likelihood weighting indeed can induce an attentional selection bias for a feature even if the respective target is invisible and that a novel target can trigger the re-shifting of attentional resources even if the novel stimulus is invisible. Thus, our findings support the view that attentional selection and consciousness can be dissociated (e.g., Lamme, 2003; Koch and Tsuchiya, 2007; Van Gaal and Lamme, 2012). They also show that the covert reallocation of feature-based attention can be studied by presenting a series of invisible targets at least if a prior selection bias had been introduced (i.e., likelihood weighting) to boost the intertrial facilitation. Therefore, our study puts forward a parsimonious methodological approach using single-item displays with masked targets and a discrimination task to examine the effects of attentional feature weighting in the absence of visual awareness. Importantly, we used volunteers’ discrimination ability to measure visual consciousness objectively but examined the effects of visual attention using discrimination response times, thereby guaranteeing a clear methodological separation between consciousness and attention.

The observed switch effect for the weighted RT model could in theory be explained by intertrial response priming. That is, the orientation perceived in the recent past (trial *n* − 1) could have primed the response to the current target (in trial *n*) so that responses speed up following repeated target orientations and slow down once a novel target is presented. This prediction is in line with our observation and challenges the attentional weighting account that we proposed to explain the effect. However, if intertrial response priming was responsible for the effect, one would expect a significant slowing of responses following a novel orientation to occur independently of the feature weighting. In other words, significant switch costs should have been observed also in the average RT model which was not the case. Intertrial response priming is contingent on awareness of the “pretarget” stimulus (e.g., Peremen et al., 2013). Accordingly, the observed switch effect should rely on pretarget AL2 and AL3 trials but not on fully unconscious pretarget trials if response priming was the underlying mechanism. To test this account, we reanalyzed the RT data using the same mixed model approach after removing those switch trials that were preceded by AL2 and AL3 pretarget trials. Importantly, the switch effect was preserved for the weighted RT model even when this time only fully unconscious trials preceded an orientation change. This finding, together with the fact that the switch effect was missing in the average RT model, makes it rather unlikely that response priming could alternatively explain the effect we observed.

## Conclusion

We demonstrated that unconscious feature changes of invisible targets can induce attentional reweighting against a prior attentional selection bias, suggesting that the shifting of attentional selection weights during the behavioral performance does not necessitate visual awareness. This finding supports previous studies stressing the dissociation of attention and visual consciousness (e.g., McCormick, 1997), however, prior studies predominantly report how unconsciously perceived cues affect shifts in spatial attention (e.g., Mulckhuyse et al., 2007). To our knowledge, this is the first study to investigate the effect of unconsciously perceived *feature* changes on visual attention. Importantly, the methodological advantage of combining subjective and objective measures of visual awareness helps to ensure that the target stimuli were truly unconsciously processed. In the next step, it will be important to shed light on the neural underpinnings supporting attentional feature-based re-weighting in the absence of visual awareness. Here, particularly the role of the frontopolar cortex (FPC) should be examined as previous findings consistently have linked it to exploratory attention shifts (for an extensive review see Mansouri et al., 2017), yet evidence showing that FPC supports attentional reallocation in the full absence of visual awareness is still missing.

## Data Availability Statement

The raw data supporting the conclusions of this article will be made available by the authors, without undue reservation. Behavioral data and scripts used for data analyses have been deposited at github, accessible via: https://github.com/LGparrot/unconscious_re-weighting_of_feature-based_attention.

## Ethics Statement

The studies involving human participants were reviewed and approved by Ethics committee Otto-von-Guericke University Magdeburg. The patients/participants provided their written informed consent to participate in this study.

## Author Contributions

LG, DS, and SP designed the study. LG and AJ collected and managed data. LG did the analysis of behavioral data, wrote all formal analysis scripts and wrote the manuscript. All authors contributed to the article and approved the submitted version.

## Conflict of Interest

The authors declare that the research was conducted in the absence of any commercial or financial relationships that could be construed as a potential conflict of interest.
