# Supplementary material for: Feature-Based Attentional Weighting and Re-weighting in the Absence of Visual Awareness
Source: Front Hum Neurosci. 2021 Jan 29;15:610347. doi: 10.3389/fnhum.2021.610347 (PMC7878679; doi:10.3389/fnhum.2021.610347)
Supplement: Supplementary file 1 [file Data_Sheet_1.docx]

**Supplementary material**

**I Detailed Trial information**

The median and the range of the number of trials for the average switch, the weighted switch, and the repeat condition for each level of subjective awareness (awareness level (AL) 1-3) are reported in the supplementary Table 1. Violin plots of the group distributions of the number of trials for each condition are depicted in Figure 1. In total 4.3 % of all trials, we obtained for the weighted switch (i.e., orientation changes away from the frequent tilt) and 5.6% for the repeat condition rated as fully unaware (AL1). 7.4% of all trials were weighted switch trials with residual awareness (AL2) and 12.6% were AL2 repeat trials. 8.5% of the trials were weighted switch trials rated as almost fully aware (AL3), while 10.9% of all trials were AL3 repeat trials. The majority of trials were obtained for the average switch condition with 10.4% of all trials rated as fully unaware, 20.5% with residual awareness, and 19.8% rated as almost fully aware.

The descriptive data clearly showed uneven proportions of trial numbers across conditions with more trials for the partially aware and almost fully aware switch and repeat condition. Thus, the means that we calculated based on sometimes only a few trials for the fully unconscious condition could have been more strongly affected by outliers and could be less reliable estimates of the true values so that the observed effect could reflect an artefact elicited by noisy data. To rule out this possibility, we already stated in the manuscript that we first repeated the analysis using a 2 SD and 2.5 SD cutoff to see if the results would be preserved with a more rigid cutoff, which was indeed the case. In addition, we conducted a control analysis in which we matched the number of trials by randomly sampling from AL2 and AL3 trials the same amount as AL1 trials and used these matched random selections per subject to repeat the mixed model analysis. The results were the same as before: we found a significant fixed effect of switch, *F*(1,33.982) = 4.4814, *p* = .0417, and awareness, *F*(2, 11.706) = 13.3356, *p* < .001. Yet, paired comparisons again showed only for AL1 trials that RTs in repeat trials were significantly faster compared RTs in switch trials, *t*(33.13) = -2.374, *p* =.0236, 95% CI [-187.5 ms, -14.4 ms], but not in AL2, *p* = .9530, nor in AL2 trials, *p* = .1753. This observation clearly contradicts the possibility that the switch effect occurred randomly due to a low number of trials. We also tested the direct relation between switch costs obtained in the weighted RT model and the number of trials aiming to prove that the effect did not depend on the number of trials obtained for individual subjects. Since this analysis required the verification of the absence of an association, we used a Bayesian based linear mixed model. First, we constructed a model with a by-subject random intercept only indicating that all variance in the observed switch costs was explained by interindividual differences only. The alternative model additionally included the number of trials obtained for switch trials for each level of awareness as a single fixed effect. Next, using the R-package “BayesFactor” and its *lmBF* function (Morey & Rouder, 2018), we calculated Bayes factors for each model and divided these two factors to obtain a BF_10_ that would favor either the null model or the model that included the number of trials as a predictor. This analysis resulted in a BF_10_ = 0.472 with anecdotal evidence rather in favor of the null model (Quintana & Williams, 2018) which suggests an association between the observed switch effect in AL1 trials for the weighted RT model and the amount of trials available for analysis in this condition was rather unlikely.

Next, we report accuracies as well as the level of subjective awareness of those trials that preceded an orientation change (i.e., “pretarget” trial information). In total 8.6% of all preceding trials were rated as subjectively fully unaware in which participants gave a correct response in the orientation discrimination task. Incorrect responses were given in 9.2% of all preceding trials rated as subjectively fully unaware. In 27.6% of all trials prior to an orientation change in which volunteers reported residual awareness of the stimulus orientation (AL2), correct responses were given, while in 7.5% of these preceding AL2 trials subjects made incorrect responses. In 29.4% of trials rated as almost fully aware (AL3) volunteers correctly discriminated the target orientation prior to a switch. Only 4% of these preceding AL3 trials were error trials. Finally, 9.7% of the trials were rated as fully aware and correct answers were given and only in 0.4% of these AL4 trials volunteers gave incorrect responses. Note that AL4 trials were not included in the analyses due to the very low number of trials. Table 2 summarizes the mean number of trials for correctly and incorrectly performed trials preceding an orientation change for each level of awareness.

**II Accuracies and signal detection analysis data**

The ability to correctly discriminate the target orientations (vertical versus non-vertical) served as the objective measure of visual awareness of the target orientation. As reported in the manuscript, we chose signal detection theory measures instead of simple accuracies (1- errors) to analyze volunteers’ task performance in the orientation discrimination task. This we did because the unbalanced design and the frequency differences of the three target orientations would have made it difficult to determine the theoretical chance level for statistical testing of the accuracies on group level. The theoretical chance level of the sensitivity (A’), however, remains unaffected by frequency differences and uneven proportions of data across conditions so that the discrimination performance on group level could be readily tested against chance using this measure. Moreover, accuracies confound the effect of sensitivity and bias on behavior, while they can be measured separately using signal detection theory (e.g., Lynn & Barret, 2014). Still, to provide a full picture of the data here, we report accuracies (1- error rates) for each condition and for the weighted and average data set.

Descriptive means of accuracies revealed an increase in task performance with increasing subjective awareness with only marginal differences between switch and repeat trials: in fully unconscious trials (AL1) the average switch model, comprising all switch trials and repeat trials, the mean accuracy was 55.93 ± 24.55% (*SD*) in repeat and 53.05 ± 18.97% in switch trials. In trials with residual visual awareness (AL2) the accuracy was 73.03 ± 13.29% in repeat and 77.10 ± 10.40% in switch trials. In trials rated as almost fully aware the mean accuracy in repeat trials was 90.39 ± 8.9% and in switch trials 88.56 ± 7.06%.

A similar pattern of behavioral performance was obtained for the weighted switch model in which the switch condition contained only orientation changes away from the heavily weighted tilt: the mean accuracy in fully unaware (AL1) weighted switch trials was 48.04 ± 17.85. For AL2 trials we found a mean accuracy of 77.97 ± 8.5% in switch trials. Again, highest accuracies were found in almost fully aware weighted switch trials (AL3) with a mean accuracy of 86.93 ± 7.01%. Note that the repeat trials in this weighted switch model were the same as in the average model. Figure 2 a) depicts boxplots of accuracies on group level for average switch and repeat trials for each level of awareness. Figure 2 b) shows accuracies on group level for the weighted switch and repeat trials for each level of awareness.

In addition to the accuracy data the average rates of hits (H), false alarms (FA), correct rejections (CR), and misses (M) for each level of subjective awareness are reported in Table 3 as well as the mean number of hit, false alarm, miss, correct rejection trials (Table 4).

**III Model selection in the LMM analysis of RT data**

To determine the final random effect structure of the LMM used to fit the RT data, we conducted likelihood ratio tests (Crainiceanu & Ruppert, 2004). First, we defined a model with visual awareness (AL1 - 3) and the change of the stimulus orientation (switch versus repeat) as the two fixed effects, we additionally added an interaction term of these two fixed effects and defined a by-subjects random intercept to account for non-independency of the data in the repeated measure within design, i.e., for baseline differences in RTs across subjects. This resulted in the following basic model: *RT ~ Awareness + Switch + Awareness:Switch + (1 | subject)*. This model was compared to a second model containing an additional by-subject random slope for awareness to model differing responses to the main factor awareness since subjects may cope differently with very low stimulus visibility. This by-subject random slope model was thus defined as follows: *RT ~ awareness + switch + awareness:switch + (1 + awareness | subject)*. Finally we defined a third alternative model with a more complex random effect structure by additionally entering a second by-subject random slope of switch to model differing response due to changes in the stimulus orientation (*RT ~ awareness + switch + awareness:switch + (1 + awareness + switch | subject)*) as well as a model containing a by-subject random slope for switch only (*RT ~ awareness + switch + awareness:switch + (1 + switch | subject)*).

Making use of the likelihood ratio test as implemented in the *anova* function of the R-package “*lme4*” (Bates, Maechler & Bolker, 2014) showed for the weighted RT data that the model containing the additional by-subject random slope for awareness significantly improved the overall model fit compared to the basic model containing only a by-subject random intercept, *X ^2^* (5, *N* = 13) = 18.525, *p* = .0023. Next, we compared the model with a by-subject random slope for awareness with a third model containing the additional by-subject random slope for switch. Here the likelihood ratio test suggested that the model with the additional by-subject random slope for switch did not better fit the RT data than the more parsimonious model containing the by-subject random slope for awareness only, *X ^2^* (4, *N* = 13) = 1.5326, *p* = .8208. Similarly comparing the model with the by-subject random slope for switch only with the basic by-subject random intercept model did not improve the overall fit, *X ^2^* (2, *N* = 13) = 0.0611, *p* = .9699. Therefore we selected the model with a by-subject random intercept and a by-subject random slope for awareness (*RT ~ awareness + switch + awareness:switch + (1 + awareness | subject)*) for hypothesis testing. In a previous study on unconscious response priming that also used a variant of the perceptual awareness scale (PAS, Ramsøy & Overgaard, 2004), a similar mixed model with an identical random effect structure was defined to analyze the unbalanced RT data (Avneon & Lamy, 2018).

We repeated the procedure outlined above also for the average RT model. Here the likelihood ratio test similarly showed that a model including a by-awareness random slope showed a better fit than the basic model with only a by-subject random intercept, *X ^2^* (5, *N* = 13) = 20.95, *p* < .001. Again, a model with an additional by-subject random slope for switch did not improve the overall fit compared to the model with only a by-subject random slope for awareness, *X ^2^* (4, *N* = 13) = 2.3028, *p* = .6803. The same was true when comparing a model with a by-subject random slope for switch to the basic by-subject random intercept model, *X ^2^* (2, *N* = 13) = 0.0508, *p* = .9749. Hence also for the average RT model we chose the model with a by-subject random intercept and a by-subject random slope for awareness for final data fitting.

**References**

Avneon, M. & Lamy, D. (2018). Reexamining unconscious response priming: A

liminal-prime paradigm. *Consciousness & Cognition, 59,* 87-103. <https://doi.org/10.1016/j.concog.2017.12.006>

Bates, D., Mächler, M., Bolker, B., & Walker, S. (2014). Fitting linear mixed-effects models using lme4. *arXiv preprint arXiv:1406.5823*.

Crainiceanu, C. M., & Ruppert, D. (2004). Likelihood ratio tests in linear mixed models with one variance component. *Journal of the Royal Statistical Society: Series B (Statistical Methodology), 66*(1), 165-185. doi:

Lynn, S. K., & Barrett, L. F. (2014). “Utilizing” signal detection theory. *Psychological Science*, *25*(9), 1663-1673.

Morey, R. D., & Rouder, J. N. (2018). BayesFactor: Computation of Bayes Factors for Common Designs. R package version 0.9.12-4.2. https://CRAN.R- project.org/package=BayesFactor.

Quintana, D. S., & Williams, D. R. (2018). Bayesian alternatives for common null-hypothesis significance tests in psychiatry: a non-technical guide using JASP. *BMC Psychiatry*, 18(1), 178. doi: 10.1186/s12888-018-1761-4

Ramsøy, T. Z., & Overgaard, M. (2004). Introspection and subliminal perception. *Phenomenology and the Cognitive Sciences*, *3*(1), 1-23. doi: 10.1023/B:PHEN.0000041900.30172.e8

**Supplementary Table 1**

*Medians and range (R = maximum - minimum) of the number of trials obtained for the switch and repeat conditions for each level of subjective awareness.*

AL1 AL2 AL3

weighted switch weighted switch weighted switch

*Median R Median R Median R*

10 87 39 59 40 78

AL1 AL2 AL3

average switch average switch average switch

*Median R Median R Median R*

26 201 90 140 83 177

AL1 AL2 AL3

repeat repeat repeat

*Median R Median R Median R*

14 98 59 87 47 107

**Note.** The *weighted* switch condition comprises only orientation changes away from the frequent to a vertical orientation or to an infrequent tilt. The *average* switch condition comprises all possible orientation changes.

**Supplementary Table 2**

*Number of trials (mean and standard error of the mean) for correct and error trials prior to an orientation change for each level of subjective awareness.*

correct AL1 correct AL2 correct AL3 correct AL4

*M SE M SE M SE M SE*

23 7 76 10 81 12 27 7

error AL1 error AL2 error AL3 error AL4

*M SE M SE M SE M SE*

25 10 21 2 11 3 1 0.4

**Supplementary Table 3**

*Confusion matrix of the signal detection analysis shows averaged rates (M) and standard errors (SEs) of hits, false alarms, correct rejections and misses for each level of subjective awareness on group level. Mean values were obtained by calculating the rates for right- and left-weighted blocks separately and consequently averaging these rates across all blocks and subjects.*

AL1

Tilted Gabor Vertical Gabor

Response non-vertical H = 0.579 ± 0.066 FA = 0.538 ± 0.066

Response vertical M = 0.421 ± 0.066 CR = 0.461 ± 0.066

AL2

Tilted Gabor Vertical Gabor

Response non-vertical H = 0.788 ± 0.029 FA = 0.261 ± 0.040

Response vertical M = 0.212 ± 0.029 CR = 0.739 ± 0.040

AL3

Tilted Gabor Vertical Gabor

Response non-vertical H = 0.849 ± 0.037 FA = 0.134 ± 0.038

Response vertical M = 0.151 ± 0.037 CR = 0.866 ± 0.038

**Note**: H = Hit rate = hits/(hits + false alarms); FA = False alarm rate = false alarms/(hits + false alarms); M = Misses rate = misses/(misses + correct rejections); CR = Correct rejection rate = correct rejections/(misses + correct rejections).

**Supplementary Table** **4**

*Average number of trials (M ±* *SE)* *of hits, misses, false alarms, and correct rejections across left- and right-weighted blocks.*

AL1

Hits Misses False Alarms Correct Rejections

(correct non-vertical) (incorrect non-vertical) (incorrect vertical) (correct vertical)

26.3 *±* 8.0 25.8 *±* 9.6 15.0 *±* 7.0 13.2 ± 5.5

AL2

90.0 ± 11.8 24.7 ± 4.6 9.9 ± 1.4 35.6 ± 5.9

AL3

84.9 ± 14.3 12.6 ± 3.8 4.2 ± 1.5 48.8 ± 8.1

**Fig. 1**: Violin plots show the number of trials for the average switch, the weighted switch, and the repeat condition as a function of subjective awareness (AL 1-3). Violin plots use density curves to depict distributions of numeric data. The width corresponds with the approximate frequency of data points in each region. The lower and upper limits of each plot is determined by the minimum and maximum value.

**Fig. 2**: Accuracies (1- error rate) for the weighted switch, and the repeat condition in a), as well as for the average switch condition b) as a function of subjective awareness (AL 1-3).
