# Supplementary figures and images for: Feature-Based Attentional Weighting and Re-weighting in the Absence of Visual Awareness
Source: Front Hum Neurosci. 2021 Jan 29;15:610347. doi: 10.3389/fnhum.2021.610347 (PMC7878679; doi:10.3389/fnhum.2021.610347)

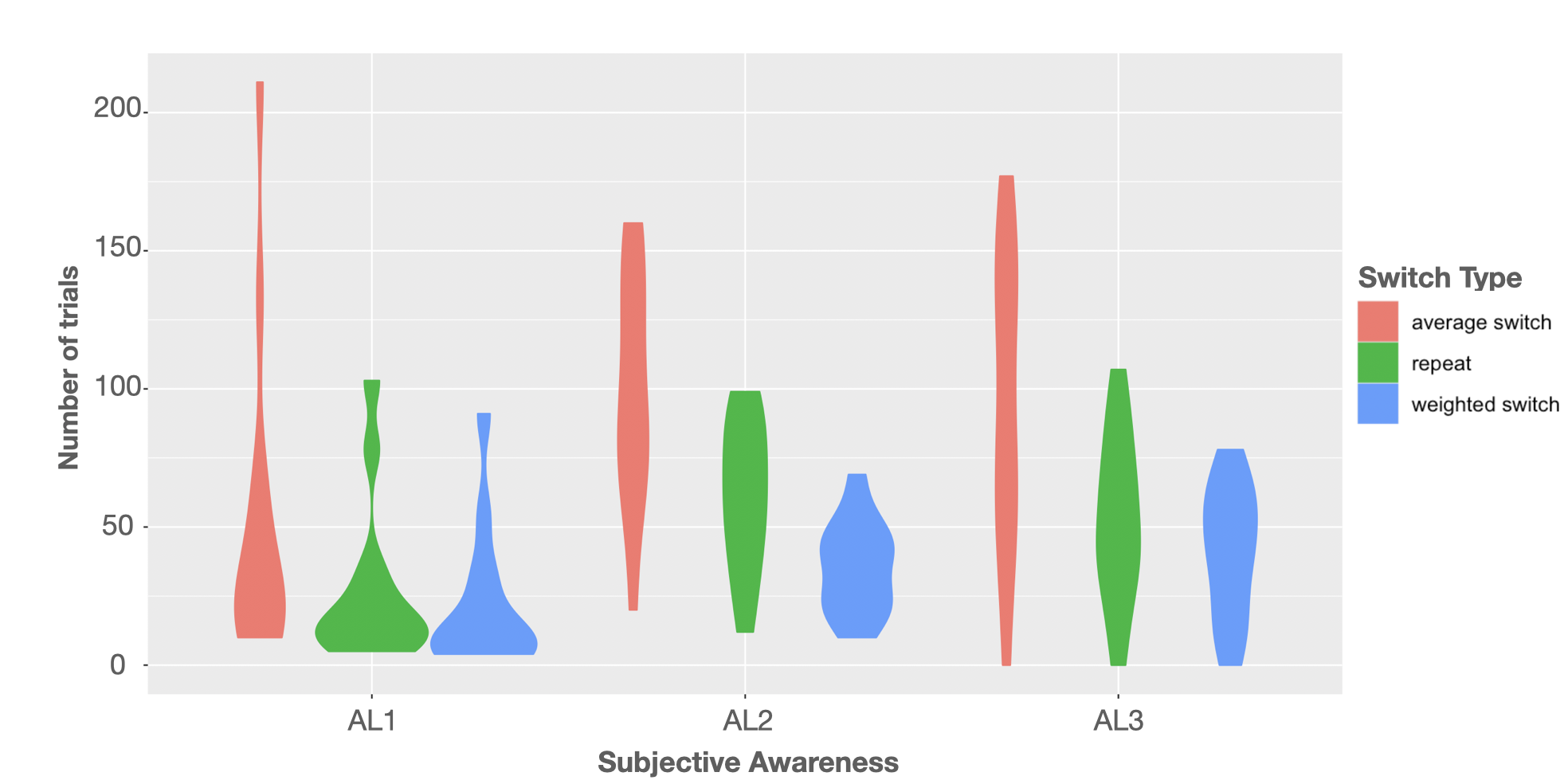

Supplement: Supplementary file 2 [file Image_1.jpeg]

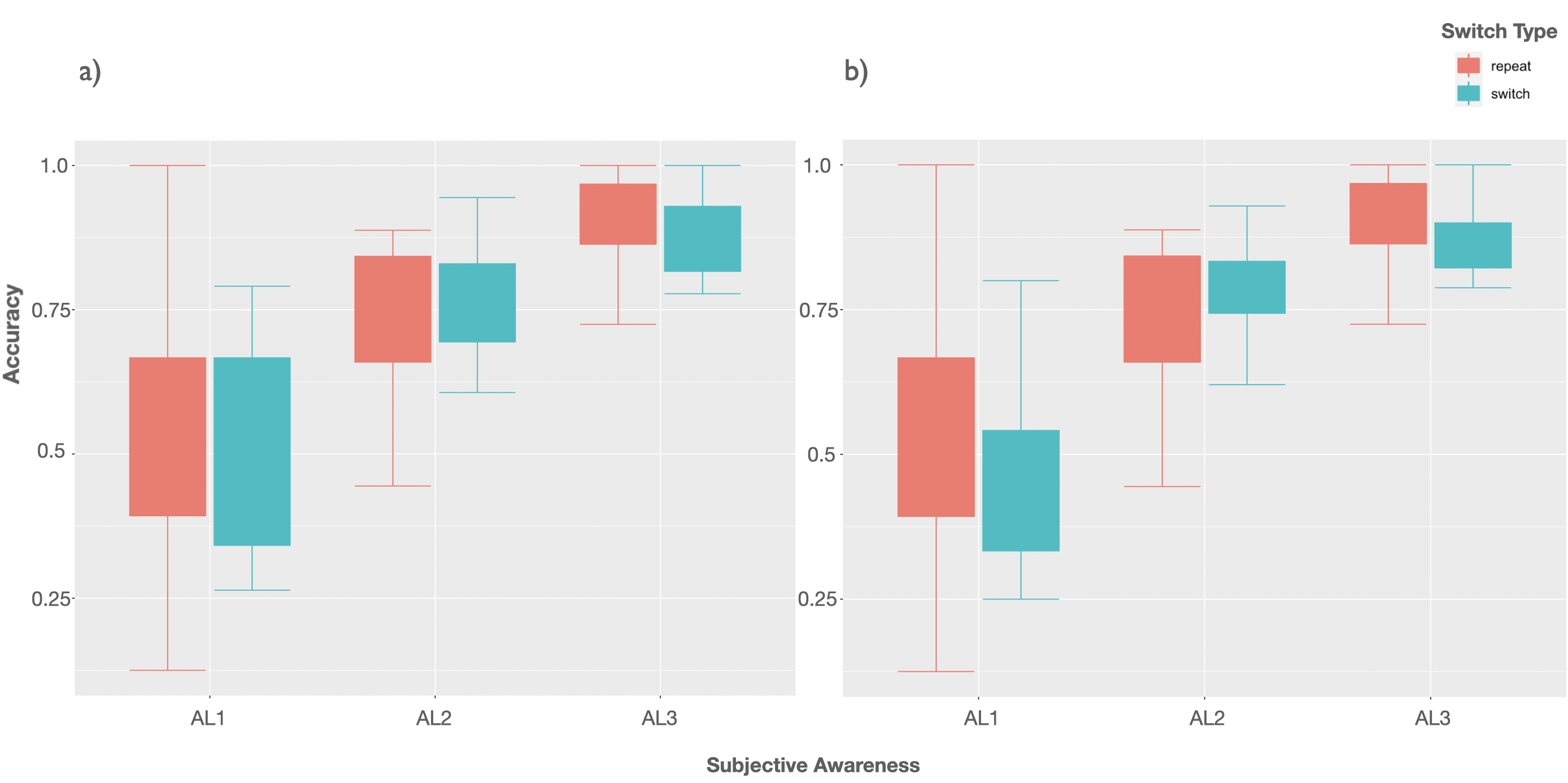

Supplement: Supplementary file 3 [file Image_2.jpeg]
